# The role of naturally-occurring canine tumors in translating conserved consequences of epithelial-mesenchymal plasticity to human cancers

**DOI:** 10.3389/fvets.2026.1802457

**Published:** 2026-04-20

**Authors:** Kimaya M. Bakhle, Anushka Dongre

**Affiliations:** Department of Biomedical and Translational Sciences, College of Veterinary Medicine, Cornell University, Ithaca, NY, United States

**Keywords:** comparative oncology, dogs, epithelial-mesenchymal transition, naturally-occurring cancer, tumor heterogeneity, tumor microenvironment

## Abstract

Epithelial-mesenchymal plasticity (EMP) is a dynamic cellular process that confers motility to epithelial cells. In carcinomas, this program advances disease progression by promoting therapy resistance, recurrent disease, and spread to distant organ sites. The mechanisms of these clinical consequences are well studied in mouse models. However, mouse models lack physiologically relevant features of human cancers, including tumor heterogeneity, immune experience, and common environmental exposures. To address this, many groups have turned to naturally-occurring cancers in companion animals. This approach, known as comparative oncology, provides a model with conserved molecular mechanisms, similar environmental and immunological exposures, and realistic tumor heterogeneity. Moreover, companion animals receive the same treatment modalities as human patients and clinical trials can be executed with reduced cost and often in a shorter time frame. Therefore, studying EMP in companion animals, such as dogs, can help identify conserved features and therapeutic vulnerabilities. Here, we review consequences of EMP in four canine cancers: mammary, prostate, squamous cell, and urothelial carcinoma, with a focus on conserved features between murine models and disease in canine and human patients.

## Introduction

The epithelial-mesenchymal transition (EMT) is a reversible, dynamic process that occurs under both physiologic and pathologic conditions (1–5). Activation of this program leads to increased motility and invasion in epithelial cells. EMT is required for embryogenesis and wound healing (6, 7). However, cancer cells can also undergo EMT, resulting in invasive primary tumors and metastatic dissemination (8–10). Once at the metastatic site, cancer cells undergo the reverse process- mesenchymal-epithelial transition (MET) to colonize the secondary organ (1, 7, 11). Moreover, EMT activation leads to a spectrum of hybrid or partial states, where cells exhibit features of both epithelial and mesenchymal cells (12–15). Due to the reversibility of this process, as well as its ability to generate hybrid states, it is being increasingly referred to as epithelial-mesenchymal plasticity (EMP) (2, 16). In cancer cells, EMP also leads to chemo- and radioresistance, stem cell-like properties, and recruitment of immunosuppressive cells (1, 15, 17–22).

EMP is well-studied in mouse models, and there is increasing evidence of this process in human tumors. Recently, studying cancer in companion animals, including dogs, for the advancement of both human and veterinary patients (a field known as comparative oncology) has gained traction (23–25). Fifty percent of older dogs develop cancer and approximately one in four dogs die from the disease, making it extremely prevalent (26). Dogs develop many of the same cancers as humans, with certain breeds demonstrating increased susceptibility to develop certain cancer types (26). Additionally, dogs are immunocompetent and share a similar environment with their owners (25). The intact and experienced immune system of dogs makes studying cancer development and treatment response more physiologically meaningful compared to immunodeficient hosts (e.g., nude and NOD *scid* gamma mice) or even immunocompetent hosts that are kept in sterile housing conditions (27, 28). The shared environment of dogs and humans allows researchers to uncover potential environmental carcinogens by studying canine cancers (29). The cancers that humans and dogs develop possess several conserved features, including histologic subtypes, driver mutations (e.g., BRAFV600E, p53, c-Kit, Bcr-Abl), transcriptional programs, metabolic pathways, and treatment modalities (e.g., chemotherapy, radiation therapy, surgery) (24, 25, 28, 30–32). The shorter lifespan of dogs compared to humans means that clinical trials of new pharmaceuticals and immunotherapies can be conducted with less cost and in a shorter time period in dogs (27). Finally, accessing samples from canine patients involves less administrative hurdles compared to human patients. For these reasons, we believe that studying the molecular features of EMP in natural-occurring canine cancers will accelerate our understanding of this cellular process, its conserved features across species, and strategies to target its consequences in patients.

In this review, we aim to identify shared features of EMP between murine models and canine cancers. We focus on four canine cancers with substantial experimental evidence of EMP: mammary, prostate, squamous cell, and urothelial carcinoma. Specifically, we highlight findings that demonstrate EMP-mediated features including migratory and invasive potential leading to metastasis, therapy resistance, stem-like properties, and immune evasion. Additionally, we identify features for which we lack experimental evidence in each cancer type. Addressing these gaps can improve our understanding of EMP in naturally-occurring tumors and how to better target this process in both veterinary and human patients.

## Malignant features of epithelial-mesenchymal plasticity (EMP)

Epithelial-mesenchymal plasticity (EMP) is a cellular program activated during development, wound healing, and carcinoma progression (1, 2, 6, 7). In all these contexts, EMP confers migratory capabilities to epithelial cells. EMP is induced by several signaling pathways including the TGF-β pathway, WNT signaling, NOTCH pathway, and activation of mitogenic growth factor receptors by their cognate ligands (e.g., EGF, FGF, HGF, PDGF) (1, 33). Additional cytokines released by immune and stromal cells within the tumor microenvironment (TME) can activate EMP, such as TNF, IL-6, and CCL18. These pathways induce expression of EMT- inducing transcription factors (EMT-TFs) (1).

EMT-TFs include Zeb1, Zeb2, Snail, Slug, Twist1, and Twist2. Different EMT-TFs are activated during different physiological contexts (development, wound healing, cancer) (1, 2). For example, in the mouse, Slug is associated with normal development of the mammary gland, whereas Snail is implicated in carcinoma progression (8). Epithelial cells normally display a cuboidal morphology, apical-basal polarity, and adherence to the surrounding epithelium and the underlying basement membrane (via cell adhesion molecules like E-cadherin, EpCAM, claudins, etc.). Activity of EMT-TFs such as Snail and Zeb1 can transcriptionally repress *CDH1* (encoding E-cadherin) and instead induce expression of vimentin and N-cadherin (34–37). These genes can be used as markers to track the progression of cells along the EMP spectrum from epithelial (high expression of E-cadherin, EpCAM, and claudins) to mesenchymal (high expression of EMT-TFs, N-cadherin, vimentin, and fibronectin) (1). Importantly, rather than operating as a binary switch, EMP is a dynamic process that can result in a multitude of hybrid or intermediate states. Within these hybrid states, cells share features and markers of both epithelial and mesenchymal cells (12–14, 38). In fact, carcinoma cells rarely transition into a completely mesenchymal state, but rather reside in a quasi-mesenchymal state where they retain some epithelial characteristics (12–14).

EMP has been shown to be involved in the progression of several cancer types including breast, pancreatic, lung, colorectal, hepatocellular, prostate and bladder cancer (1, 2, 39–57). In carcinoma cells, EMP activation results in several malignant features: (1) invasive and migratory capabilities, leading to metastatic dissemination, (2) resistance to multiple therapeutic approaches, (3) stem-like properties, and (4) immunosuppression, resulting in immunotherapy resistance (8–10, 15, 17, 18, 20–22, 58–62). EMP is probably most notable for its role in invasion of the surrounding tissue, resulting in metastasis (referred to as the invasion-metastasis cascade). This is a multistep process, where carcinoma cells from the primary tumor invade the surrounding stroma, intravasate into blood or lymphatic vessels, travel in circulation to nearby lymph node or distant organ site, extravasate from the vessel, and colonize the secondary tissue (1, 63–67). EMP is involved in each step of this process to different extents. Snail and Zeb2 activate expression of matrix metalloproteinases (MMPs), which allow cells to degrade the surrounding basement membrane and stroma to promote cell invasion (68, 69). In circulation, tumor cells retain E-cadherin expression and exist in a hybrid state, to facilitate formation of circulating tumor cell clusters (66, 70, 71). Finally, at the site of metastasis, cells undergo a mesenchymal-epithelial transition to form the secondary tumor (11, 67).

Another consequence of EMP is acquired resistance to therapies, including chemotherapy and radiation therapy (50, 72). This occurs through regulation of genes involved in cell death, drug efflux, and stem cell maintenance (1, 20, 22). Particularly, these resistant, mesenchymal-like cells show increased resistance to p53-mediated apoptosis (22). Additionally, EMP-induced cancer stem cell upregulate ATP-binding cassette (ABC) transporters that promote drug efflux (1, 19). Acquisition of therapy resistance is closely tied to stem-like properties upon EMP activation. Cancer stem cells are multipotent and have increased self-renewal capacity. Cells residing in a hybrid state in particular, display stem-cell properties (8, 13–15, 17, 18, 21, 22, 43). EMP-induced cancer stem cells likely contribute to post-treatment residual disease that drives disease relapse and metastasis (73).

Finally, and most recently, EMP has been linked to immunosuppression in carcinomas. EMP activation in breast cancer leads to an immunosuppressive TME, as well as resistance to immunotherapies (58, 62, 74, 75). Specifically, EMP modulation has been shown to regulate function of both innate and adaptive immune cells (59, 61, 74, 76–78). Finally, EMP controls expression of cancer cell-intrinsic immunoregulatory molecules (60, 74).

Many of these impactful studies leading to discoveries about EMP function in carcinomas have been conducted using mouse models. Our ability to carefully manipulate the signaling pathways and cellular plasticity of cancer cells is essential for these mechanistic studies. To test the relevance of these findings for human patients, we propose studying features of EMP in naturally-occurring canine carcinomas.

## Comparative oncology as an avenue to advance EMP research

To assess the translational implications of EMP for human cancers, we can make use of naturally-occurring canine tumors. Dogs and human tumors share similarities in tumor biology including cellular morphology, heterogeneity, metastasis, recurrence, and treatment response (25). At the molecular level, canine and human cancers share driver mutations, transcriptional signatures, and metabolic alterations. Additionally, the unique breed structure of dogs, along with the increased susceptibility of specific cancers in certain breeds poses an advantage for uncovering the genetic drivers of tumorigenesis (26, 79). Finally, like humans, dogs have a fully functional and experienced immune system, which is important for understanding tumor-immune interactions (80). In dogs, mammary, prostate, oral squamous cell, and urothelial carcinomas possess the most evidence of EMP and therefore will be the focus of this review (Table 1).

**Table 1:** Key characteristics, experimental approaches, and therapeutic targets of epithelial-mesenchymal plasticity in canine tumors.

Associated transcriptional programs	Cell line models	Experimental readouts	Clinically observable correlates	Therapeutic targets and strategies
Stem cell properties (98, 104) Drug resistance (110–113, 115) Hypoxia (113) WNT signaling (113) Upregulation of immunosuppressive factors (98, 104)	E20, E37, M5, M25 (118, 162) CHMp, CHMm (115) CHMp13a (116) CF41 (117, 118) Drug-resistant cell lines with EMP and CSC signatures: CMT-7364 (115) REM134 (111–113) TAMp and TAMm (115)	Immunohistochemistry/Immunofluorescent labeling (99–101, 104–107, 110, 113, 115, 117, 118) (RT-PCR/RNA-seq) (98, 101, 104, 110, 111, 113, 115, 117–119) Immunoblotting (110, 111, 113, 115, 116, 118) Tumorsphere/colony-forming capacity (111, 113, 115, 118) Invasion and migration assays (110, 111, 113, 115, 117, 118) Tumor growth and metastasis in xenograft models (110, 117)	Decreased survival (98–100) Larger tumor size (100) Increased histologic grade (100, 104) Increased mitotic index (100) Metastasis (100, 105, 117)	Inhibit TGF-β1 (117) Revert cells to more epithelial state with metformin (117) Target CD109 (104)
Stem cell properties (136)	PC1 and PC2 (123) TihoDProAdcarc1258, TihoDProAdcarc0846, TihoDProAdcarc1508, TihoDProAdcarc1511.1, TihoDProMetadcarc1511.2, TihoDProMetadcarc1511.3 (136) 1,508, 1,258, Leo (134) Ace-1 (141, 142) LuMA (136)	Immunohistochemistry (121–126) RT-PCR/RNA-seq (123, 125, 130, 134–136) Pyrosequencing methylation analysis (123, 125) Immunoblotting (123, 130, 134, 136) Colony-forming assay (134) Invasion and migration assays (134–136) Tumor growth and invasion in a xenograft model (135, 136)	Decreased survival (123) Increased Gleason score (123) Metastasis (123, 124)	Inhibit GRPr to revert cells to a more epithelial state (135)
Upregulation of immune checkpoint molecules and immunosuppressive factors (95, 104, 137, 139, 144)	SCC1 and CoSCC (95) TSCCLN#5, TSCCLN#6, oSCC-4 (150, 151)	Immunohistochemistry/Immunofluorescent labeling (137, 138, 141–144) RT-PCR/RNA-seq (95, 104, 137, 140, 144) Spatial transcriptomics (139) Immunoblotting (143, 144) Invasion and migration assays (143, 144)	Metastasis (141)	Immune checkpoints PD-L1 and CTLA-4 (145) Inhibit IL-6 (151)
Basal subtype (150) Upregulation of immune checkpoint molecules and immunosuppressive factors (104, 146, 150) Hypoxia (150)	TihoDProCarc/TCC0840, TihoDProTCC1509, TihoDUrtTCC1506 (130) K9TCC-PU-AxA, K9TCC-PU-Sh, K9TCC, K9TCC-PU-Nk (149)	Immunohistochemistry (147–150) RT-PCR/RNA-seq (146, 150) Immunoblotting (150)	Decreased survival (147) Metastasis (148, 149)	Immune checkpoint PD-1 (146, 150) MTA1 (149)

EMP, Epithelial-mesenchymal plasticity; EMT-TF, Epithelial-mesenchymal transition transcription factor; CSC, Cancer stem cell; RT-PCR, Real-time polymerase chain reaction; GRPr, Gastrin-releasing peptide receptor; MTA1, Metastasis-associated protein 1.

Breast cancer is the most common cancer in women and accounts for 32% of all new diagnoses (81). Human breast cancers can be classified by expression of hormone receptors (estrogen and progesterone receptors) and HER2 expression. Triple-negative breast cancer (TNBC) lacks expression of all three receptors and is the most aggressive form of breast cancer, accounting for 10–15% of cases (82). TNBCs activate the EMP program, leading to high rates of metastasis and chemotherapeutic resistance (52, 83–85). In dogs, mammary tumors represent 50–70% of cancers diagnosed in intact female dogs and about 50% of these tumors are malignant and metastasize to distant organs. Delayed spaying increases risk of mammary tumor development, suggesting that tumorigenesis is impacted by hormones (86). Canine mammary tumors usually contain mixtures of luminal epithelial, myoepithelial, and mesenchymal cells, suggesting they may be a good model for the heterogeneity observed in human breast cancers (86, 87). Canine mammary tumors share several other features with human breast cancers including histologic and molecular subtypes, clinical features, and metabolic alterations (31). Finally, canine mammary tumors are diagnosed by histopathology, staged using the tumor, lymph node, metastasis (TNM) system, and primarily treated by surgery. Patients with metastatic disease or vascular/lymphatic invasion are often treated with adjuvant radio- or chemotherapy (86).

Prostate carcinoma is the most common cancer in men, with increasing incidence of metastasis (81, 88, 89). Other than humans, dogs are the only large mammal with a significant incidence of spontaneous prostate cancer. These two species also share similarities in the anatomical structure of the prostate (30). Unlike in humans, androgens play a minor role in canine prostate cancer, as evidenced by the increased incidence of prostate cancer in castrated dogs (30, 90). Moreover, prostate cancer in dogs is more aggressive and displays local invasion, as well as widespread metastasis, particularly to the bone (30). Canine prostate cancer is diagnosed by imaging, cytology, or histopathology. Dogs bearing prostate tumors are treated through surgery, radiotherapy, chemotherapy, as well as other emerging interventional oncology approaches (91) This indicates that canine prostate carcinoma may be a good model for advanced, metastatic, and hormone-non-responsive prostate cancer in humans (30, 92).

Canine and human oral squamous cell carcinomas are both locally invasive and have potential to metastasize to the lymph nodes (93, 94). Additionally, these two species share a similar copy-number alteration landscape (94). Molecular studies comparing canine oral squamous cell carcinoma and human head and neck squamous cell carcinoma has revealed homologous transcriptional signatures between the two species, including EMT, cell cycle progression, and upregulation of the immune-inhibitory molecules PD-L1 and CTLA-4 (95). Squamous cell carcinoma (SCC) is the second most common skin malignancy in the United States. In humans, only 3% of cases metastasize (96). In dogs, oral squamous cell carcinoma (OSCC) is the second most common oral malignant tumor. Canine OSCC shares similar incidence with human head and neck cancers. Canine OSCC is locally invasive and metastatic, and local disease recurrence is common (94). Canine oral squamous cell carcinoma is primarily treated by surgical excision. Radiotherapy and chemotherapy are used in patients with recurrent, metastatic, or inoperable tumors (97).

Cancer of the urinary bladder accounted for an estimated 84,870 new cases last year and is the fourth most common cancer in males (81). In humans, urothelial carcinoma (also known as transitional cell carcinoma) cases can be divided into a more common, non-invasive form and less common, high-grade muscle-invasive form. In dogs, invasive urothelial carcinoma (InvUC) accounts for about 1.5–2% of all naturally-occurring cancers and more than 90% of bladder cancer cases (32). Dogs of certain breeds (Scottish Terriers, West Highland White Terriers, and Beagles) demonstrate a higher risk of developing. In both dogs and humans, InvUC locally invades the bladder wall and similar displays metastatic rates (32). In dogs, InvUC is diagnosed through histopathology and treated primarily with chemotherapy, as the location of InvUC lesions often prevents surgical excision (32). All four of these canine cancer types possess substantial evidence for EMP activation. Due to the similarities between canine and human tumors, these studies may have implications for the role of EMP in human cancers.

## Evidence for EMP in canine carcinomas

### Mammary carcinoma

**Figure 1:**
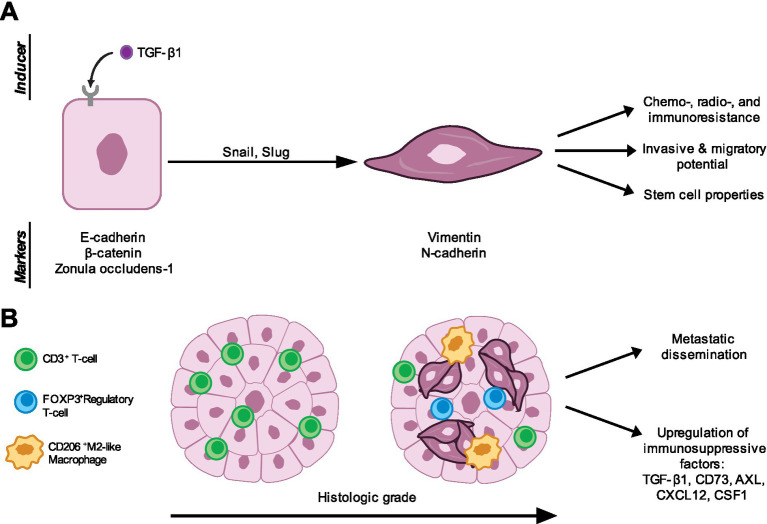
Properties of epithelial-mesenchymal plasticity (EMP) activation in canine mammary carcinoma (CMC) cells and alterations to the tumor microenvironment. **(A)** Findings from multiple groups support EMP activation in CMC samples and cell lines. TGF-β1 has been established as an inducer of EMP in CMC cells, likely resulting in activation of epithelial-mesenchymal transition transcription factors (EMT-TFs), including Snail and Slug (84, 106, 111, 117). Progression along the EMP spectrum has been tracked in CMC cells with the epithelial markers E-cadherin, β-catenin, and zonula occludens-1 and mesenchymal markers vimentin and N-cadherin (99–101, 104, 105). EMP activation has been shown to result in resistance to multiple forms of therapy including chemotherapy, radiotherapy, and immunotherapy (110–113, 115, 118, 119). Moreover, quasi-mesenchymal CMC cells gain invasive and migratory potential, as well as stem cell properties. **(B)** CMCs of advanced histologic grade have been shown to activate EMP (104, 105). EMP was also associated with metastatic dissemination in CMCs (105, 107). Activation of EMP also regulates immune infiltration into the tumor microenvironment (TME) (98). CMC samples containing predominantly epithelial cells recruited higher numbers of CD3^+^ T-cells. Conversely, CMCs containing quasi-mesenchymal cells recruited fewer CD3^+^ T-cells and instead more regulatory T-cells and M2-like macrophages (104). Finally, quasi-mesenchymal CMCs upregulated several immunosuppressive factors including TGF-β1, CD73, AXL, CXCL12, and CSF1 (98, 104).

The role of EMP in canine mammary tumors (CMTs) has been explored by multiple groups. Transcriptomic profiling by Kim et al. (98) has revealed that a subset of CMTs activate EMT and show upregulation of Snail, Slug, Zeb1, Zeb2, and TGF-β1, as well as downregulation of claudin genes and E-cadherin. Dogs with tumors displaying this EMT signature showed decreased survival. Yoshida et al. (99) showed that loss of E-cadherin and the tight junction protein Zonula occludens-1 and gain of N-cadherin and vimentin was observed more frequently in malignant CMTs than benign ones. Additionally, they found that loss of E-cadherin was associated with a low one-year survival rate. Gama et al. (100) showed that reduced E-cadherin and *β*-catenin was associated with shorter overall and disease-free survival. They also showed that reduced E-cadherin expression was associated with larger tumors, high histologic and invasion grades, high mitotic index, and metastasis. Yu et al. (101) have shown that malignant CMTs upregulate N-cadherin and vimentin and downregulate E-cadherin compared to benign tumors and healthy tissue (Figure 1A). Similar to human breast cancers, CMTs are graded on a I-III scale, based on morphological features including degree of tubule formation, nuclear pleomorphism, and mitotic index (102). In both species, increased histologic grade is associated with poor prognosis (102, 103). We have shown that high-grade canine mammary carcinomas (CMCs; a subtype of CMTs of epithelial origin) activate EMP. Specifically, cancer cells display a spindle-shaped cellular morphology, cytoplasmic translocation of E-cadherin, and upregulation of vimentin. The co-expression of E-cadherin and vimentin may suggest that the cells reside in a hybrid state (104). Work by Raposo-Ferreira et al. supports these findings, as they showed that the percentage of cells co-expressing E-cadherin and vimentin was significantly higher in grade II and III CMTs compared to grade I tumors. Interestingly, there were more E-cadherin^+^ Vimentin^−^ and E-cadherin^+^ Vimentin^+^ cancer cells in distant metastases compared to primary tumors, as well as increased Snail/Slug expression in primary tumors (105) (Figure 1B). This could be suggestive of a MET that leads to formation of metastases.

Several EMT-TFs have been implicated in CMT malignancy. Sammarco et al. showed that grade II CMTs upregulate Snail, but not Slug, Zeb1, or Zeb2 compared to healthy mammary gland tissue and grade I CMTs. They also showed cytoplasmic translocation of E-cadherin, which negatively correlated with luminal cell markers CK8 and CK18 (84). Cheon & Kim showed that Slug may also play a role in CMT malignancy, as Slug expression was increased in malignant CMTs compared to benign tumors and normal mammary gland tissue. Moreover, Slug expression was associated with increased cell proliferation, larger tumor size, metastasis, and reduced overall and disease-free survival (106). Gamba et al. found that E-cadherin expression decreased with advanced, invasive disease and metastasis in invasive micropapillary carcinoma samples. Snail and Twist expression was negatively correlated with E-cadherin expression. Lymph node metastasis also showed upregulation of Zeb1, with concurrent E-cadherin expression (107) (Figure 1A). In human breast cancers, Snail and Slug have been associated with tumor dedifferentiation and lymph node metastasis (57, 108, 109).

Multiple labs have established drug-resistant mammary cancer cells and in doing so, identified connections between EMP and cancer stem cells. Zhou et al. demonstrated that doxorubicin-resistant CMT-7364 cells increased expression of vimentin and decreased expression of E-cadherin, suggesting EMT activation. These drug-resistant cells also showed increased migratory and invasive capacity *in vitro* (110). Pang et al. and Penzo et al. have shown that EMT is associated with increased stem cell activity, as well as chemotherapy, radiation therapy, and immunotherapy resistance using the CMC cell line REM134. Specifically, they showed that a subpopulation of CMC cells has tumorsphere-forming capabilities and these cells upregulate the stem cell markers Oct4 and Nanog, as well as EMT markers fibronectin and Twist, accompanied by loss of E-cadherin and *β*-catenin (111). Tumorsphere-forming cells displayed increased resistance to the chemotherapeutic agent doxorubicin, ionizing radiation, and recombinant feline interferon-*ω* treatment compared to adherent cells (111, 112). Gray et al. (113) established radioresistant (RR) human breast cancer (MCF-7, ZR-751, MDA-MB-231) and CMT (REM-134) cells, which display upregulation of EMT- and hypoxia-related genes. RR MDA-MD-231 cells did not show upregulation of these genes, which could be because these cells represent TNBC and display EMP (114). RR human and canine cells showed increased migratory and invasive potential *in vitro* and enhanced WNT signaling (113). Tamoxifen-resistant CMT cells established by Xu et al. (115) also show increased migration and potential EMT activation, based on E-cadherin loss and Zeb1 gain (Figure 1A).

Pang et al. (111) also showed that treatment of adherent CMC cells with TGF-β was sufficient to induce EMT, as well as enhanced cell migration and tumorsphere-forming ability. Work by Yoshida et al. (116) supports that TGF-β induces EMT in CMT cells, using the cell line CHMp13a. Consistent with these findings, Leonel et al. showed that targeting TGF-β with short hairpin RNA (shRNA) knockdown or metformin treatment reduced N-cadherin and E-cadherin in CMT cell line CF41. This MET resulted in reduced migratory capacity *in vitro* and metastasis *in vivo* (117) (Figure 1).

Increased tumorsphere-forming ability in treatment-resistant CMT cells provides evidence for stem cell activity associated with EMT (111). For example, Xavier et al. (118) also showed that mesenchymal-like CMT cell lines expressed lower E-cadherin and higher Zeb1 and Zeb2, which correlated with tumorsphere-forming capacity. Additionally, Kim et al. (119) showed that metastatic CMTs express higher levels of Oct4, Sox2, and Nanog and a nonsignificant decrease in E-cadherin expression compared to nonmetastatic CMTs (Figure 1A).

Using CIBERSORT to estimate immune cell proportions using bulk RNA sequencing data, Kim et al. (98) found that CMTs with EMT activation had reduced CD8^+^ T-cells and increased M2-like macrophages. Our group has shown that high-grade, quasi-mesenchymal CMCs recruit increased proportions of FOXP3^+^ regulatory T-cells and CD206^+^ M2-like macrophages, as well as fewer CD3^+^ T-cells. We also showed that heterogeneous tumors containing mixtures of epithelial and quasi-mesenchymal cells recruited increased numbers of FOXP3^+^ regulatory T-cells and CD206^+^ M2-like macrophages, suggesting that just a proportion of cancer cells activating EMP could be sufficient to result in an immunosuppressive TME (104). We found that heterogeneous and quasi-mesenchymal CMCs upregulated immunosuppressive factors including TGF-β and CD73 (104). Additionally, using RNA sequencing data published by Kim et al., we identified associations between EMP and upregulation of several immunosuppressive factors including AXL, CXCL12, and CSF1 (104, 120) (Figure 1B).

Based on the studies described here, there is substantial evidence for EMP activation in canine mammary carcinomas. In CMCs, this process is characterized by loss of E-cadherin, β-catenin, and other junctional proteins, as well as upregulation of N-cadherin and vimentin. Additionally, multiple groups have observed co-expression of E-cadherin and vimentin, suggesting the presence of hybrid states in CMCs. Studies support the involvement of multiple EMT-TFs in CMCs including Snail, Slug, and Zeb1. The involvement of these different EMT-TFs may depend on the stage of disease. However, this is largely based on mRNA upregulation and further work is required to understand the functional role of these EMT-TFs in CMCs. Multiple groups have shown that TGF-β induces EMP *in vitro*. There is also ample evidence that EMP is associated with resistance to multiple treatment modalities including chemotherapy, radiation therapy, and immunotherapy. This therapy resistance may be related to the ability of EMP to generate stem-like cells. Finally, there is emerging evidence to suggest that EMP is associated with increased pro-tumor immune cells within the TME, as well as upregulation of immunosuppressive factors in CMCs. These findings support a role for EMP in advancing metastatic disease, therapy resistance, and immunosuppression in human breast cancers.

### Prostate carcinoma

**Figure 2:**
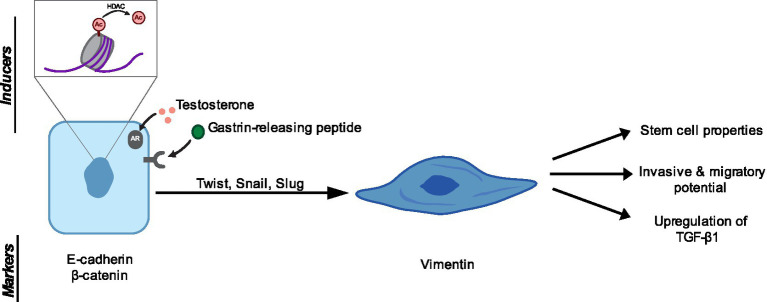
Regulators of EMP in canine prostate carcinoma and resulting phenotypes. Several inducers of EMP have been identified in canine prostate carcinoma. Namely, histone deacetylase (HDAC) activity, activation of androgen receptors, and gastrin-releasing peptide have been associated with EMP (134–136). The epithelial-mesenchymal transcription factors (EMT-TFs) Twist, Snail, and Slug have been implicated in canine prostate carcinoma (135, 136). E-cadherin and β-catenin have been established as epithelial markers and vimentin has been used as a mesenchymal marker (121–126). Finally, EMP induction results in stem cell properties, invasive and migratory potential, and upregulation of the immunosuppressive cytokine TGF-β1 in canine prostate carcinoma cells (92, 130, 134, 135).

Multiple groups have shown evidence of EMP in canine prostate carcinoma. Fonseca-Alves et al. showed that prostate carcinoma samples gained expression of vimentin, decreased expression of E-cadherin, and showed translocation of *β*-catenin from the membrane to the cytoplasm and nucleus (121–123). Increased vimentin expression in prostate carcinoma samples and metastases was supported by Rodrigues et al. (124). Kobayashi et al. (125) also found cytoplasmic and nuclear translocation of β-catenin, as well as cytoplasmic translocation of E-cadherin. Lean et al. (126) observed cytoplasmic and nuclear translocation of β-catenin, as well as vimentin-positive luminal cells in prostatic carcinoma samples (Figure 2). Human and canine prostate carcinomas are graded using the Gleason system, which indicates differentiation status and is predictive of disease progression (30). Fonseca-Alves et al. showed that loss of E-cadherin is associated with shorter survival time and higher Gleason scores. Moreover, they found that E-cadherin expression was regulated by methylation of the *CDH1* gene. Interestingly, prostate cancer metastases showed high expression of E-cadherin, suggesting that these cells regain E-cadherin expression upon metastasis, which could be indicative of a MET program (123).

Lai et al. found that castration led to increased appearance of less-differentiated growth patterns in their prostatic carcinoma samples. Like the mammary gland, the prostate is made up of a layer of CK8/18^+^ luminal cells with an underlying layer of CK5/14^+^ basal cells. Lai et al. found that tumor samples had increased proportions of CK5^+^ and CK14^+^ cells, suggesting less-differentiated tumor cells (92). This luminal-to-basal transition may reflect an EMT program, however some view these as two independent processes (52, 127–129). Interestingly, castrated dogs had greater proportions of CK14^+^ cells, which may suggest that these androgen-independent tumors contain a higher content of stem cells (92).

Packeiser et al. (130) showed that canine prostate cancer cell lines with high vimentin expression and low expression of epithelial and luminal (CK8/18) markers had higher invasive potential. In human prostate cancer cell lines, androgen receptor (AR) signaling restoration reduces cell migration and invasion (131–133). Vasilatis et al. (134) investigated whether this is also true in canine prostate cancer cells. They found conflicting effects on cell migration with AR restoration, with some cell lines showing decreased migration and others showing increased migration. AR restoration resulted in upregulation of EMT markers Snail, vimentin, and N-cadherin in two out of three cell lines, and increased invasion in one of these cell lines. Knockdown of vimentin in these cells reduced migration, suggesting that AR restoration in these cells leads to EMT and increased vimentin-dependent invasion (134) (Figure 2). The variability in migratory and invasive effects upon AR restoration among these cell lines reflects the heterogeneity of prostate carcinoma and potentially may reflect different stages of disease.

A study by Elshafae et al. (135) suggests that gastrin-releasing peptide receptor (GRPr) activity may be a driver of EMT in canine prostate cancer. GRPr was upregulated in prostate cancer samples compared to normal prostates. Treatment with a GRPr agonist increased cell proliferation and migration of GRPr-overexpressing Ace-1 (canine prostate carcinoma) cells *in vitro*. Ace-1 cells overexpressing GRPr and treated with the GRPr agonist also upregulated EMT-TFs Twist, Snail, and Slug and the mesenchymal marker Vimentin, and downregulated both E-cadherin and *β*-catenin (Figure 2). Treatment with a GRPr antagonist induced an MET with downregulation of vimentin and Snail and upregulation of E-cadherin. *In vivo,* Ace-1 cells overexpressing GRPr and treated with GRPr agonist formed larger tumors with spindle-shaped morphology that invaded into the surrounding muscle and adipose tissue. Implantation of these GRPr overexpressing Ace-1 cells into the tibia resulted in larger tumors with osteolytic pattern, when compared to bone tumors formed by Ace-1 cells (135). This study provides mechanistic evidence that GRPr activity induces EMT, resulting in more aggressive tumor behavior *in vivo.*

Another study by Elshafae et al. (136) investigated the role of a histone deacetylase inhibitor (HDACi) on Ace-1 cells. Treatment with the HDACi reduced cell migration and invasion *in vitro.* Ace-1 cells treated with the HDACi showed decreased expression of E-cadherin, N-cadherin, Twist, upregulation of Zeb1, Snail, and Slug, and no change in the expression of vimentin and *β*-catenin. Expression of these markers is somewhat discordant, as E-cadherin levels would usually be expected to be inversely related to N-cadherin and Twist levels. However, the decrease in E-cadherin and increase in certain EMT-TFs may suggest an EMT. These results are also based on mRNA levels, which may not reflect protein levels. In mouse models and cell lines, EMT has been shown to generate stem cell properties (17, 18, 21). In Elshafae’s study, treatment with the HDACi also increased mRNA expression of some prostate stem cell markers, including CD44, DDX5, and ITGA5. By flow cytometry, surface CD44 expression was slightly increased with HDACi treatment. *In vivo,* HDACi treatment reduced tumor incidence and metastasis, as well as bone lysis within the tibia. HDACi-treated mice did show increased CD44 immunolabeling in the tumors compared to control mice (136). These results suggest that histone deacetylase (HDAC) activity may control EMP and stem cell activity in canine prostate carcinoma.

There are limited studies investigating the connection between EMP and immune cell infiltration and activity in canine prostate carcinoma. In the Elshafae et al. (135) study, Ace-1 cells induced to undergo EMT by GRPr overactivity showed upregulation of TGF-β1, which may suggest upregulation of at least one immunosuppressive factor (Figure 2).

Taken together, there is strong evidence that canine prostate carcinoma activates EMP, which promotes invasion and potentially metastasis. EMP in prostate carcinoma is characterized by cytoplasmic translocation of E-cadherin and nuclear translocation of β-catenin, as well as increased expression of EMT-TFs and vimentin. Increased proportions of CK5/14^+^ basal cells may also indicate EMP activation. EMP may also be associated with stem cell activity in canine prostate carcinoma, but this requires further investigation. EMP seems to be regulated by epigenetic modifications (methylation of *CDH1* and HDAC activity) and GRPr activity. An important distinction between human and canine prostate carcinoma is the involvement of hormones, as castration actually increases prostate cancer incidence in dogs. AR restoration may be a therapeutic strategy, but this has shown heterogeneous effects in canine prostate cancer cell lines and has not been tested *in vivo*. Finally, the interplay between EMP and immune response should be further investigated, but findings of TGF-β1 upregulation by Elshafae et al. may suggest an immunosuppressive role.

### Squamous cell carcinoma

**Figure 3:**
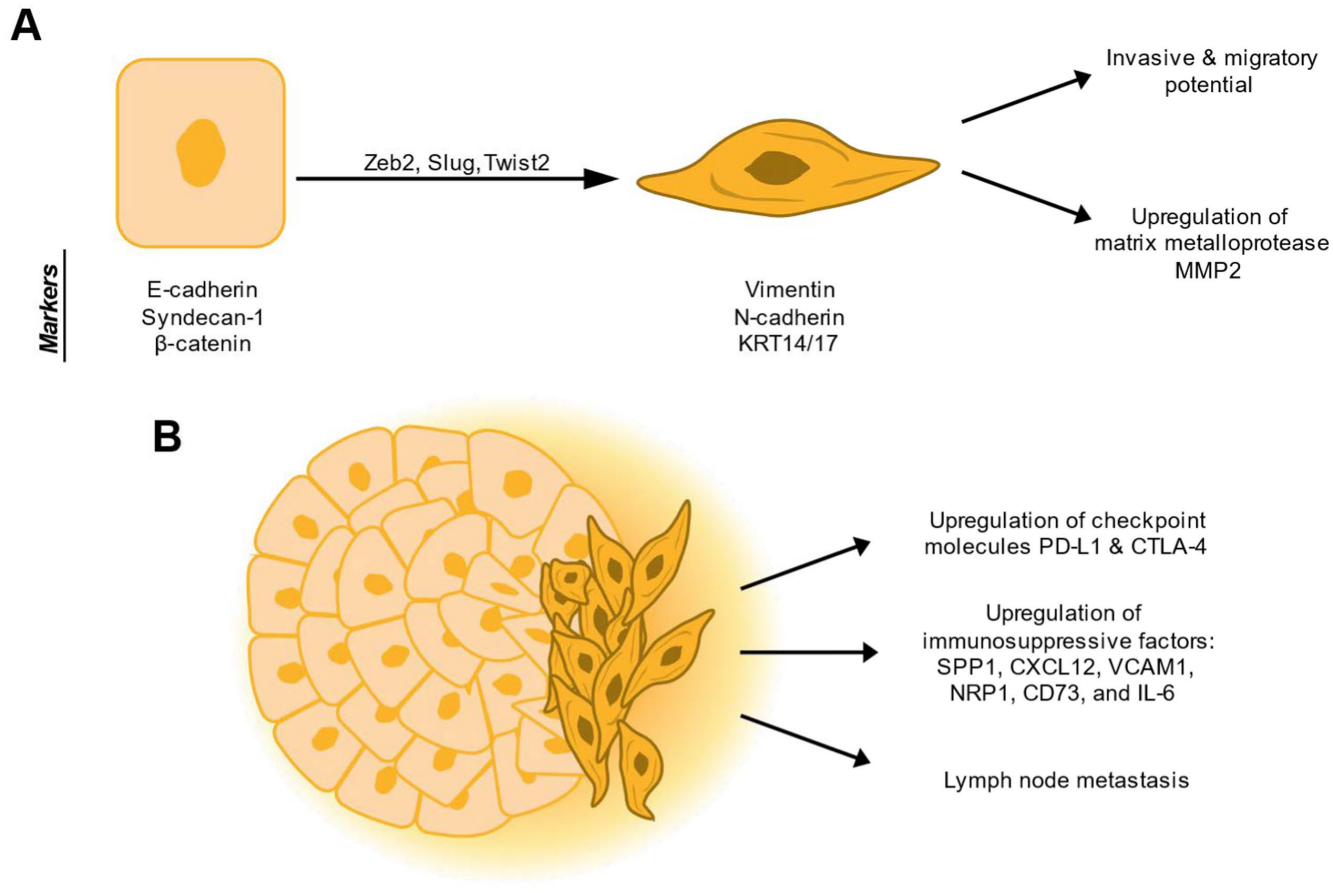
Epithelial-mesenchymal plasticity (EMP) at the invasive front of canine oral squamous cell carcinoma (OSCC). **(A)** EMP in canine OSCC has been suggested to be driven by epithelial-mesenchymal transcription factors (EMT-TFs) Zeb2, Slug, and Twist2 (95, 139, 143). In canine OSCC cells, EMP activation has been measured using epithelial markers E-cadherin, syndecan-1, and β-catenin and mesenchymal markers Vimentin, N-cadherin, and KRT14/17 (137, 138, 140–142). EMP results in invasive and migratory potential as well as upregulation of the matrix metalloprotease MMP2 (140, 143). **(B)** In canine OSCC, EMP is most frequently observed at the invasive front of the tumors (137, 139, 141). In this region, EMP is associated with upregulation of the immune checkpoint molecules PD-L1 and CTLA-4 and immunosuppressive factors including SPP1, CXCL12, VCAM1, NRP1, CD73, and IL-6. Finally, EMP results in lymph node metastasis in canine OSCC (95, 104, 137, 139, 142, 144).

A study by Liu et al. identified that canine SCC samples altered the same pathways as human head and neck squamous cell carcinomas. Specifically, several genes related to TGF-β signaling and EMT-TFs Twist1 and Snail were overexpressed in tumor samples compared to normal tissue. EMT activation in these tumors was confirmed by immunofluorescent staining (94). A study by Peralta et al. also used RNA sequencing to show that the EMT gene signature was enriched in OSCC samples compared to healthy gingiva. They confirmed this by immunohistochemistry, showing that the invasive front of the tumors displayed loss of cytokeratin expression and gain of vimentin (137). Similarly, Tabtieang et al. (138) found that both canine and human OSCC samples downregulated E-cadherin and the cell adhesion molecule syndecan-1 compared with normal controls. Guscetti et al. (95) also found evidence for EMT by RNA sequencing of laser-capture microdissected tumor and matched normal epithelial from OSCC samples. Spatial transcriptomic profiling by Goldschmidt et al. showed enrichment of the EMT pathway in surface cancer, deep tumor, and peritumoral dysplastic tissue compared to normal tissue. This molecular signature was driven by upregulation of Zeb2, Slug, Twist2 in surface and deep tumor (Figure 3A). In peritumoral dysplasia, the EMT signature was driven by COL1A1, COL1A2, and COL3A31 (139). Finally, Pisamai et al. (140) showed that OSCC samples downregulated cell adhesion molecules, including E-cadherin, and upregulated the matrix metalloprotease MMP2.

Nagamine et al. (141) observed that cells at the invasive front of OSCC samples show downregulation of E-cadherin, *β*-catenin, and desmoglein, and upregulation of vimentin and N-cadherin by immunohistochemistry. Mestrinho et al. showed that OSCC samples of increased histologic grade display cytoplasmic translocation of E-cadherin. Samples with lymph node metastasis also showed cytoplasmic translocation of E-cadherin (142) (Figure 3B).

Noguchi et al. showed that EMT may be a Slug-driven process in OSCC, as this EMT-TF is overexpressed in tumor tissue compared to normal tissue. Overexpression of Slug in OSCC and tonsillar squamous cell carcinoma cells increased Snail and Vimentin expression and decreased E-cadherin expression. Moreover, OSCC and tonsillar SCC cells overexpressing Slug showed increased invasion and migration *in vitro*. These effects were reversed with knockdown of Slug (143). Conversely, work by Guscetti et al. (95) identified a partial state driven by ZEB2 activity, with upregulation of Krt14 and Krt17, without upregulation of other EMT-TFs. This was supported by spatial transcriptomics data published by Goldschmidt et al. as well (139) (Figure 3A).

EMT activation in OSCC has been associated with increased PD-L1 and CTLA-4 expression (95). Using data published by Peralta et al., we found that several immunosuppressive factors were associated with EMT markers in canine OSCC samples including SPP1, CXCL12, VCAM1, NRP1, and CD73 (104, 137). In the Goldschmidt et al. study, SPP1 was found to be upregulated in surface cancer tissue compared to both normal and peritumoral dysplastic tissue. They also found that the macrophage score was negatively correlated with E-cadherin and positively correlated with vimentin expression (139). Finally, they found that expression of CTLA-4, TIM-3, LAG3, PD-1, PD-L2, and TIGIT were increased in the tumor microenvironment compared to normal samples (Figure 3B).

Noguchi and Shimonishi showed that non-tonsillar OSCC cell lines and tissues, as well as tonsillar OSCC tissues, upregulated IL-6 compared to normal mucosa tissue. Dogs with tonsillar OSCC with high expression of IL-6 showed decreased survival. Treatment with recombinant IL-6 increased cell migration and invasion and induced vimentin expression in certain tonsillar OSCC cell lines (144) (Figure 3B).

Multiple independent studies have shown that OSCC samples activate EMP with loss of cell adhesion molecules like E-cadherin and increased expression of EMT-TFs such as Slug and Zeb2. This results in gain of vimentin and cytoplasmic translocation of E-cadherin, as we have observed in canine mammary carcinomas, which is suggestive of a hybrid EMP state. This EMP signature was frequently observed at the invasive front of the tumor. Transcriptomic profiling has enabled researchers to identify associations between EMT and immunosuppression, as evidenced by upregulation of inhibitory and exhaustion markers, as well as immunosuppressive factors, particularly SPP1 and IL-6. OSCC has relatively low incidence of metastasis, suggesting the EMP activation may lead to local invasion and recurrence, but not metastatic dissemination in the case of OSCC. Moreover, EMP has not been associated with treatment resistance or stem-like properties in OSCC.

### Urothelial carcinoma

**Figure 4:**
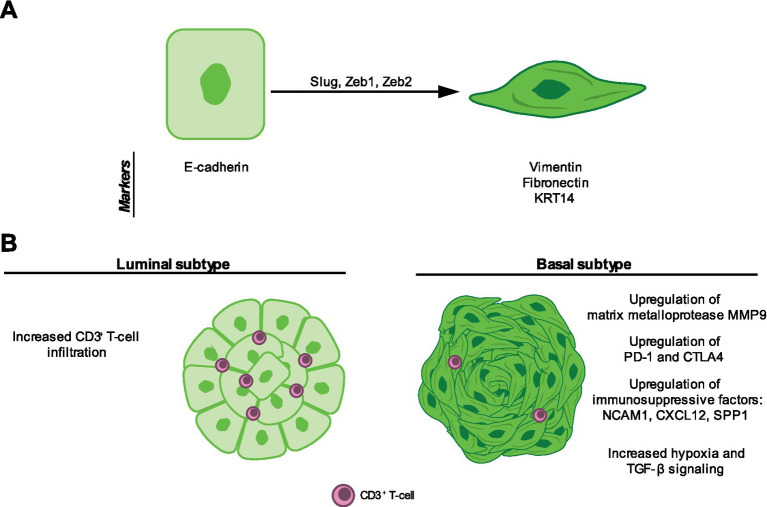
The basal subtype of canine invasive urothelial carcinoma (InvUC) activates epithelial-mesenchymal plasticity (EMP). **(A)** In canine InvUC cells, EMP is activated by epithelial-mesenchymal transcription factors (EMT-TFs) Slug, Zeb1, and Zeb2 (146). EMP has been tracked using the epithelial marker E-cadherin and mesenchymal markers vimentin, fibronectin, and KRT14 (146, 147). **(B)** Canine InvUC can be divided into luminal and basal subtypes, of which the basal subtypes displays EMP activation (32, 146). Basal InvUC also shows several immunosuppressive changes including decreased T-cell infiltration, upregulation of immune checkpoint molecules PD-1 and CTLA-4, and increased expression of immunosuppressive factors NCAM1, CXCL12, and SPP1 (104, 146, 150, 157). Finally, basal InvUC upregulates the matrix metalloprotease MMP9 and displays increased hypoxia and TGF-β signaling (146, 150).

At the molecular level, human InvUC segregates into basal and luminal subtypes, similar to breast cancer. Basal InvUC samples express EMT markers (32, 145). Dhawan et al. have identified that canine InvUC shares these same luminal and basal subtypes, with the basal subtype upregulating EMT markers (Figure 4B). Specifically, luminal tumors displayed high expression of E-cadherin, whereas basal tumors upregulated Krt14, Slug, Zeb1, and Zeb2 (146) (Figure 4A). Another study by Shinada et al. showed that canine urothelial carcinoma samples displayed vimentin and fibronectin labeling in tumor cells by immunohistochemistry. They observed that some cells co-expressed E-cadherin and vimentin, suggesting that these cells may reside in a hybrid state (12–14). Additionally, they showed that dogs bearing tumors with increased vimentin expression had reduced median survival time (147). Another study showed that plasmacytoid and rhabdoid variants of urothelial carcinoma is also associated with EMP. These three cases also showed aggressive spread to the peritoneum and other metastatic sites (148). This may suggest the EMP activation in canine urothelial carcinoma is associated with more aggressive biological behavior and as a result, reduced survival.

The study by Dhawan et al. also showed that basal InvUC samples upregulated MMP9 (Figure 4B). Upregulation of matrix metalloproteases is associated with EMP and allows tumor cells to invade surrounding stroma (146). Campanelli et al. (149) showed that tumors displaying local muscle invasion downregulated E-cadherin compared to tumors without invasion, suggesting EMP activation. Interestingly, they also observed that lung metastatic lesions upregulated E-cadherin compared to primary tumors. Together, this may argue that canine urothelial carcinoma cells undergo EMT to invade the surrounding muscle and initiate metastasis and then undergo MET to colonize the metastatic site.

Contrary to these findings, a study by Cronise et al. showed an enrichment of EMT markers in normal bladder tissue compared to urothelial carcinoma samples, however the sample size of this study was rather small. Additionally, they found that several cell adhesion molecules were downregulated in tumor tissue compared to normal, including ITGA5, ITGA9, and CADM3 (150).

Basal InvUC samples have been shown to upregulate PD-1 (146). This marker can indicate T-cell activation or exhaustion, depending on the context and co-expression of other inhibitory molecules (151–154). These results also come from bulk RNA sequencing, so the source of PD1 expression is unknown. In the Cronise et al. study, they found upregulation of both PD-1 and CTLA-4, which is more indicative of a dysregulated T-cell phenotype (150). Moreover, they found that tumors with low numbers of infiltrating CD3^+^ T-cells showed enrichment of genes related to TGF-β signaling and hypoxia, both of which are implicated in activation of EMP (33, 62, 155, 156). Using data generated by Sommer et al., we identified associations between expression of EMT markers with three immunosuppressive paracrine factors: NCAM1, CXCL12, and SPP1 (104, 157) (Figure 4B).

Together, these studies use a combination of transcriptomic analysis and histology to provide evidence of EMP activation in InvUC. This process is marked by reduced E-cadherin expression and gain of vimentin. These cells demonstrate locally invasive and metastatic behavior, with a potential MET program that facilitates metastasis. There has been some evidence that InvUC, particularly the basal subtype, may be immunosuppressive, but this requires further investigation. Moreover, there is little to no evidence that EMP is associated with stem cell-like properties or therapy resistance in urothelial carcinoma.

## Therapeutic potential and limitations of EMP-associated targets

As we have detailed above, canine mammary, prostate, squamous cell, and urothelial carcinomas share several EMP-associated malignant features (Table 1). Briefly, this includes therapy resistance, acquisition of stem cell properties, and immunosuppression. Clinically, this manifests as larger tumor size, metastasis, and decreased survival. These studies have also identified potential therapeutic targets that are associated with EMP markers and/or poor prognosis. In both OSCC and urothelial carcinoma, recent findings have shown that more-mesenchymal tumors upregulate immune checkpoints PD-1, PD-L1, and CTLA-4 (95, 139, 146, 150). This provides clear rationale for use of immune checkpoint blockade therapy for patients with tumors that display EMP activation. In fact, immune checkpoint blockade therapy has shown promise for dogs with InvUC and *in situ* tumor vaccines have been effective in dogs with head and neck cancer (80, 158, 159). However, the upregulation of additional immunosuppressive factors such as TGF-β1, SPP1, and IL-6 may necessitate combination therapy approaches that target both immune checkpoint molecules and EMP-regulated immunosuppressive factors (95, 104, 137, 139, 144, 150). Another therapeutic approach in mammary and prostate cancer cells is to revert the EMP-activated cancer cells to a more epithelial state. In mammary cancer cells, this was achieved through a combination of TGF-β1 silencing and metformin treatment (117). In prostate cancer cells, this was done by inhibiting the GRPr (135). Such an approach is advantageous because it may simultaneously decrease expression of several EMP-regulated genes related to invasion, drug resistance, stemness, and immunosuppression. However, the dynamic nature of EMP poses a risk, as not all cells will revert to the same EMP state, and this can generate additional tumor heterogeneity.

In addition to these pan-cancer approaches, several cancer-specific targets have also been identified. We recently determined that the expression of the glycoprotein CD109 is closely tied to activation of EMP in mammary carcinomas. This association was also observed in murine mammary carcinomas and human breast cancers, further supporting the potential of CD109 as a biomarker or therapeutic target across species (104). Campanelli et al. showed that MTA1 is upregulated in UC samples and negatively correlated with E-cadherin expression. Lung metastases showed a further increase in MTA1 (149). These studies are examples of how studying the molecular mechanisms of EMP in canine cancers can reveal therapeutic targets that have potential for clinical trials in veterinary patients.

However, translating these EMP-associated targets to the clinic would not be without challenges. Firstly, identifying EMP activation in a tumor would require a core biopsy to identify cellular morphology and expression of EMP markers. This means these EMP-associated targets would likely not be used in the neoadjuvant setting, unless a non-invasive approach (i.e., liquid biopsy) can be developed to confirm EMP activation in tumor cells (160, 161). Additionally, EMP is a temporally dynamic process, which again is difficult to monitor non-invasively. This means that taking a biopsy at the single timepoint does not represent the spectrum of EMP states that may eventually emerge within the tumor. Furthermore, since EMP is activated later in disease progression, the primary tumor may have already metastasized once EMP activation is detected. When considering therapeutic targets, expression of the target molecule should also be validated in both primary and metastatic lesions, as these sites may have a distinct spectrum of EMP states and therefore heterogeneous expression of the therapeutic target. Differential response of the primary tumor and metastases should also be considered when determining the endpoint of the clinical trial. For therapeutic agents that aim to revert more-mesenchymal cells to a more-epithelial state, biospecimens should be ideally collected throughout the treatment course to measure expression of EMP markers and cellular morphology. For immunology-based approaches, the lack of canine-specific antibodies may be a hurdle for measuring immune cell infiltration as a metric of treatment response. Based on the ability of EMP to promote therapy resistance, the most effective therapeutic option may be one that targets these resistance mechanisms in combination with the current standard-of-care regimen. Despite these limitations, appropriately-designed clinical trials would have high potential to provide clinical utility of EMP-associated targets to veterinary and human patients.

## Conclusion

EMP is a dynamic, reversible program that occurs in advanced stages of carcinoma progression (1, 2, 7). Studies conducted in murine models have detailed the ability of this program to promote invasion and metastasis, therapy resistance, stem-like properties, and immunosuppression across multiple cancer types. However, in many cases, whether these findings can be translated to human cancers remains unknown. Naturally-occurring cancers in companion animals, such as dogs, are a physiologically-relevant model of human cancers. In dogs, mammary, prostate, oral squamous cell, and urothelial carcinoma possess the most evidence for EMP activation. In CMCs, increased activity of Snail, Slug, and Zeb1 has been identified, leading to hybrid epithelial-mesenchymal states. Multiple groups have generated therapy-resistant CMC cells, which demonstrate properties of stem cells. However, it is unclear whether treatment enriches for a pre-existing population of more-mesenchymal cells that are intrinsically treatment resistant or activates EMP in treatment-naïve cells, leading to acquired resistant. Among these four tumor types, CMCs show the most evidence for an association between EMP and immunosuppression. In prostate carcinoma, EMP activation is evidenced by change in expression of characteristic epithelial and mesenchymal markers, as well as cytokeratins associated with luminal and basal cells. Studies in canine prostate cancer cell lines has also revealed roles for HDACs and GRPr in modulating EMP. The role of hormones should be considered when comparing mammary and prostate carcinoma in canine and human patients, as dogs are frequently spayed or neutered. Slug and Zeb2 have been implicated in EMP of OSCC, and a potential partial state has been identified. Multiple studies have identified SPP1 and IL-6 to be associated with EMP in OSCC. Additional immunosuppressive factors, as well as expression of inhibitory receptors and exhaustion markers have also been connected with EMP. Finally, in urothelial carcinoma, EMP has been associated with invasion and metastasis. Several studies have also suggested T-cell dysregulation related to EMP in urothelial carcinoma.

These findings provide a strong foundation for bridging the translational gap between mechanistic EMP studies in murine models and improving outcomes for veterinary and human patients. However, there are still several drawbacks left to be addressed in this area. Firstly, the use of archived patient samples eliminates the temporal dynamics of EMP in these samples. In reality, cancer cells are constantly transitioning between more-epithelial and more-mesenchymal states, in response to microenvironmental cues. By using archived samples, we are only observing a snapshot of that dynamic process. Additionally, formalin-fixed paraffin-embedded (FFPE) sections contain only a sample of the tumor and may not accurately represent the whole tumor. This is particularly relevant as we have shown that minority fractions of more-mesenchymal cancer cells are sufficient to drive treatment resistance (74). Furthermore, measuring EMP markers by bulk RNA sequencing (RNA-seq) of tumors can be confounded by the expression of mesenchymal markers by stromal cells. Measuring bulk mRNA or protein levels or either cell lines or tumors also fails to capture the potential heterogeneity of partial/hybrid EMP states present within a sample. Technologies like single-cell/nucleus RNA-seq and spatial transcriptomics provide greater resolution and therefore can generate a more meaningful analysis of the spectrum of EMP states within a given tumor. Even with these current limitations, naturally-occurring canine tumors present a valuable translational model to promote the development of targets based on therapeutic targets. Future studies should make use of clinical information associated with patient samples, cell lines identified here, as well as technologies like spatial transcriptomics to simultaneously profile multiple markers of EMP and its functional and clinical outcomes. Doing so will accelerate the application of our knowledge on EMP-mediated processes to improvements in clinical outcomes for both veterinary and human patients.
